# Oxidation Behavior of Carbon Fibers in Ceramizable Phenolic Resin Matrix Composites at Elevated Temperatures

**DOI:** 10.3390/polym14142785

**Published:** 2022-07-07

**Authors:** Tingli Yang, Chuang Dong, Yiyang Rong, Zongyi Deng, Pengfei Li, Pengkun Han, Minxian Shi, Zhixiong Huang

**Affiliations:** Key Lab of Advanced Technology for Specially Functional Materials, Ministry of Education, School of Materials Science and Engineering, Wuhan University of Technology, Wuhan 430070, China; 257416@whut.edu.cn (T.Y.); dongchuang@whut.edu.cn (C.D.); rongyiyang0808@163.com (Y.R.); zong_yi_deng@whut.edu.cn (Z.D.); 303773@whut.edu.cn (P.L.); hpk051157@whut.edu.cn (P.H.); zhixiongh@whut.edu.cn (Z.H.)

**Keywords:** carbon fiber, oxidation, ceramizable, polymer–matrix composites, high-temperature properties, ablation

## Abstract

Carbon fiber fabric-reinforced phenolic resin composites are widely used as thermal protection materials for thermal protection systems in hypersonic vehicles and capsules. In this work, carbon fiber fabric-reinforced boron phenolic resin composites modified with MoSi_2_ and B_4_C were prepared via a compression molding technique. The high-temperature performance of the composites as well as the oxidation behavior of the carbon fibers was studied. The results indicate that the incorporation of B_4_C improves the performance of composites at high temperatures. The residual weight rate of composites with 15 phr B_4_C (BP-15) sufficiently increased from 23.03% to 32.91% compared with the composites without B_4_C (BP-0). After being treated at 1400 °C for 15 min, the flexural strength of BP-15 increased by 17.79% compared with BP-0. Compared with BP-0, the line ablation rate and mass ablation rate of BP-15 were reduced by 53.96% and 1.56%, respectively. In addition, MoSi_2_ and B_4_C particles had a positive effect on the oxidation of carbon fibers in the composites. After treatment at 1400 °C, the diameter of the as-received carbon fiber was reduced by 31.68%, while the diameter of the carbon fiber in BP-0 and BP-15 decreased by 15.12% and 6.14%, respectively. At high temperatures, the liquid B_2_O_3_ from B_4_C and MoSi_2_-derived complex-phase ceramics (MoB, MoB_2_, Mo_2_C, Mo_4.8_Si_3_C_0.6_) acted as an oxygen barrier, effectively mitigating the oxidation degree of the carbon fibers.

## 1. Introduction

During hypersonic flight in the atmosphere, the thermal load transmitted to a spacecraft due to the phenomenon of aerodynamic heating has a serious impact on the operation of the instruments in the cabin and jeopardizes the safety of the spacecraft [1]. As a result, a thermal protection system (TPS) must be installed on the surface of the spacecraft to prevent heat from entering the interior of the cabin and ensure the spacecraft’s normal operation. Common thermal protection is generally achieved by use of a heat sink, ablation, and radiation, of which ablative thermal protection is the safest and most cost-effective thermal protection scheme. Ablative materials achieve thermal protection through their own ablation processes. On the one hand, physical and chemical changes such as melting, decomposition, and sublimation occur in ablated materials at high temperatures, which causes a large amount of heat to be dissipated. On the other hand, the carbon layer formed by the cracking of the material surface can further prevent heat transfer to the interior of the material [2,3].

Fiber-reinforced resin matrix composites with designability and simple processes have been used in different industries, especially in the field of ablative thermal protection materials. Among a variety of resin matrix materials, phenolic ablation materials are widely used as ablation materials because of their excellent heat resistance and thermal stability [4]. However, phenolic resin is brittle and easy to crack at high temperatures, forming a loose and porous carbon layer that further accelerates the oxidative cracking of the internal structure and reduces the residual strength of the material [5]. In order to improve the performance of phenolic materials in high-temperature applications, phenolic resins are modified by adding ceramic fillers to obtain ceramizable composites, which allows the material to be molded at low temperatures and exhibit excellent properties at high temperatures. The introduction of ceramic fillers leads to the ceramizable reaction of composites at high temperatures, and the formation of dense ceramic structure on the surface plays a role in thermal oxygen resistance, which improves the high-temperature performance of composites. Aqeel Saghar et al. [6] demonstrated that the ablation rate of hybrid carbon fiber phenolic matrix composites with 5 wt% silicon carbide added was reduced to 33%. Shuang Wang et al. [7] explained that the introduction of silicon carbide particles effectively improved the mechanical properties, thermal properties, and ablation resistance of carbon–phenolic (C/Ph) composites. Jiuqiang Song et al. [8] found that the addition of ZrSi_2_ increased the initial decomposition temperature of the silicone rubber composites and reduced the linear and mass ablation rates. Yaxi Chen et al. [9] found that the method of introducing zirconium diboride particles could significantly improve the ablation resistance and insulation performance of C-Ph composites. Jie Ding et al. [10] showed that the addition of titanium diboride enhanced the thermal stability of the phenolic resin and the mechanical performance of carbon–phenolic composites at high temperature. Feng Xu et al. [11] found that TaSi_2_/ZrSi_2_ modified carbon–phenolic composites possessed a good resistance to long-term laser ablation. Wei Yang et al. [12] revealed that the addition of MoSi_2_ reduced the linear and the mass ablation rate of composites by about 94.46% and 39.09%. Among them, MoSi_2_ had excellent oxidation resistance at high temperatures. JianHui Yan et al. [13] prepared a protective MoSi_2_ coating on a Nb substrate, which exhibited excellent oxidation resistance in air at 1200 °C. However, when the temperature was lower than 800 °C, the creep resistance of MoSi_2_ became poor and insect degradation occurred [14]. As the temperature increased, the oxidation resistance of MoSi_2_ increased [15,16,17]. The introduction of B_4_C has been found to effectively improve the oxidation resistance of MoSi_2_ at relatively low temperatures. Ping Zhang et al. [18] prepared MoSi_2_-B_4_C coatings on Nb alloy via a spark plasma sintering (SPS) process. His work found that the MoSi_2_-B_4_C coating showed better oxidation resistance at 1450 °C compared to the single-layer MoSi_2_ coating.

Carbon fiber is widely used as a reinforcement fiber in thermal protection ablation materials, as it has low density (1.7–2.0 g/cm^3^), high strength, high modulus (tensile strength 3–7 GPa, tensile modulus 200–650 GPa), a low thermal expansion coefficient (0–1.1 × 10^−6^/K), high thermal conductivity (10–160 W/m∙K) and other excellent performance [19,20,21,22]. Carbon fiber can still maintain good mechanical properties in an inert atmosphere above 2000 °C. However, it is easily oxidized under a high-temperature aerobic environment, which greatly weakens its comprehensive mechanical properties, and the stability of the composites declines sharply [23,24]. Gyungha Kim et al. [25] explored the mechanical properties of carbon fibers after heat treatment in nitrogen and oxygen atmospheres. They found that the tensile strength at 500 °C was equivalent to that of the virgin carbon fibers in the nitrogen atmosphere, decreasing to 71% at 1000 °C. In the oxygen atmosphere, the tensile properties gradually decreased at 500 °C and the carbon fibers deteriorated above 600 °C, at which point the tensile properties were unable to be measured. Therefore, solving the oxidation problem is the key to the use of carbon fiber-reinforced composites for high temperatures [26,27,28]. Feng Xu et al. [29] prepared ZrSiO_4_ sol and sprayed it on carbon fiber fabrics, improving the interfacial properties and the ablation resistance of the composites. Liuyang Duan et al. [30] introduced polycarbosilane (PCS) as an interface layer onto carbon fiber, which effectively improved the performance of carbon fiber-reinforced phenolic resin composites (CPR) at high temperatures. Zhenyue Zou et al. [31] showed that ZrO_2f_-coated C_f_ hybrid fibrous reinforcements exhibited good thermal stability and high oxidation resistance. Guangyuan Yang et al. [32] proposed two kinds of carbon fibers with antioxidant coating, which were used as reinforcement components to prepare phenolic resin matrix composites. The results showed that both SiC/SiO_2_ coated carbon fibers and SiC-ZrO_2_-MoSi_2_/Ni coated carbon fibers improved the oxidation resistance of the composites without reducing the mechanical properties of the composites, with the failure temperature at around 1200 °C to 1600 °C.

Many studies have shown that the interface modification between fibers and substrates as well as the introduction of fillers have a positive effect on the high-temperature performance of composites. However, little attention has been devoted to the oxidation behavior of carbon fibers in ceramizable composites. Therefore, the purpose of this work is to study the performance of ceramizable composites and the oxidation behavior of carbon fibers in ceramizable composites at high temperatures. Carbon fiber fabric-reinforced boron phenolic resin composites modified by MoSi_2_ and B_4_C were prepared via a compression molding technique. The micromorphology of both the carbon fibers and the composites as well as the phase composition, thermal stability, flexural strength, and ablation resistance of the composites were characterized. This provides theoretical guidance for further research on the performance of carbon fiber and its composites in high-temperature environments.

## 2. Experimental

### 2.1. Raw Materials

Carbon fiber fabric (W-7022FF-400, Weihai Guangwei Composite Materials Co., Ltd., Weihai, China) was used for the reinforcement of composites while boron phenolic resin (THC-400, Shaanxi Taihang Fire Resistant Polymer Co., Ltd., Xian, China) was used as the matrix. MoSi_2_ with a particle size of 1 μm, used as filler, was supplied by Shanghai Buwei Applied Materials Technology Co., Ltd., China while B_4_C with a particle size of 1–10 μm, used as a fluxing agent, was obtained from Shanghai Aladdin Bio-Chem Technology Co., Ltd., Shanghai, China. Absolute ethanol (analytically pure, Sinopharm Chemical Reagent Co., Ltd., Ningbo, China) was used as a solvent. All materials were used as-received without further purification.

### 2.2. Preparation of Composites 

**Figure 1:**
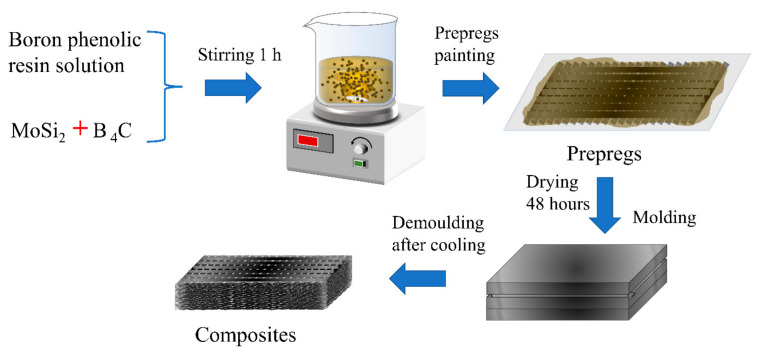
Preparation process of composites.

The preparation of composites followed the following procedures (Figure 1). Firstly, boron phenolic resin powder was dissolved in an equal mass of ethanol at 80 °C for 4 h. Then, MoSi_2_ and B_4_C particles were introduced into boron phenolic resin solution according to the formula in Table 1 and mechanically stirred for 1 h at a speed of 600 rpm to obtain a uniformly mixed ceramizable boron phenolic resin solution. Next, the ceramizable boron phenolic resin solution was coated onto carbon fiber fabric to prepare prepregs with the mass ratio of carbon fiber fabric to solution being 1:2.5. The prepregs were dried for 48 h at 35 °C to sufficiently evaporate the ethanol before being piled. Finally, the piled prepregs were put into a mold and cured in a plate vulcanizing machine via a compression molding technique to obtain ceramizable composites [33]. 

### 2.3. Thermal Oxidation Treatment of Carbon Fibers and Composites

The as-received carbon fiber fabric was treated in an air atmosphere in a muffle furnace for 15 min. The temperatures were 800 °C, 1000 °C, 1200 °C, and 1400 °C, respectively. The same went for flexural strength test samples before being tested in a universal testing machine.

### 2.4. Characterizations 

A scanning electron microscope (JSM-7500F, Tokyo, Japan) was used to observe the microscopic morphology and elemental composition of samples after high-temperature oxidation treatment. After high-temperature treatment, the composites were subjected to phase analysis using an X-ray diffractometer (D/MAX-RB, The Hague, Netherlands) at a scan rate of 10°/min. The thermal stability of the carbon fibers and composites was characterized using an integrated thermal analyzer (STA449F3) from room temperature to 1450 °C with a heating rate of 10 °C/min. The density of the composites was tested using the Archimedes method. The flexural strength of composites was tested via a universal testing machine (RGM4100, Chengdu, China) according to the Chinese recommended standard GB/T 1449-2005. The functional groups of the carbon fibers were recorded using a Fourier transform infrared spectrometer (Nexus, Gaithersburg, MD, USA) in the range of 4000 to 500 cm^−1^ while the degree of graphitization was calculated based on the Raman spectra recorded using a Raman microprobe (InVia, Wotton under Edge, UK). The ablation performance of the composites was measured under an oxygen–acetylene torch according to the Chinese standard GJB 323A-96. The line ablation rate and mass ablation rate were calculated based on the following formulas:

(1) $$\mathrm{Line}\;\mathrm{ablation}\;\mathrm{rate}=\frac{\Delta l}{\Delta t}$$

(2) $$\mathrm{Mass}\;\mathrm{ablation}\;\mathrm{rate}=\frac{\Delta m}{\Delta t}$$

where Δl and Δm represent the change in thickness and the mass loss of samples, respectively, and Δt stands for change in time.

## 3. Results and Discussion

### 3.1. Thermal Stability of Carbon Fibers and Composites

**Figure 2:**
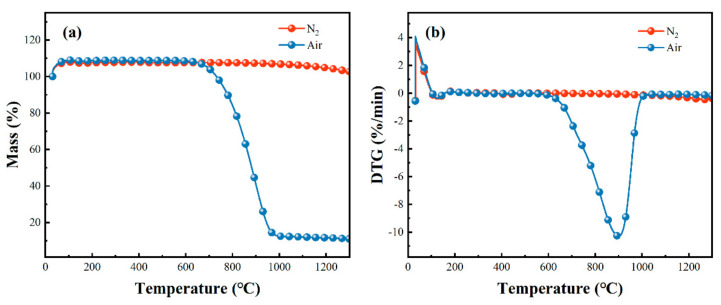
TG curve (**a**) and DTG curve (**b**) of carbon fibers in nitrogen and air atmospheres.

Thermogravimetric analysis was used to analyze the thermal stability of the carbon fibers and composites in nitrogen and air atmospheres, as shown in Figure 2. There was no obvious weight change in the carbon fibers in the nitrogen atmosphere. The slight peak at around 60 °C in the DTG curve in the nitrogen atmosphere was attributed to desorption of absorbed air and water. However, the weight loss increased significantly from 600 °C to 1000 °C in the air atmosphere, and we noted an obvious peak at around 900 °C. The final residue yield was only around 15% above 1000 °C, which was much different from that of carbon fibers in the nitrogen atmosphere. The above-mentioned results show that carbon fibers are easy oxidize above 600 °C in an air atmosphere.

**Figure 3:**
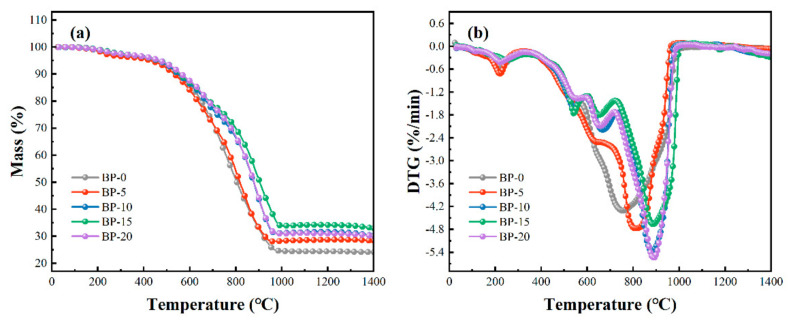
TG curve (**a**) and DTG curve (**b**) of MoSi_2_ particles modified boron phenolic resin composites with different contents of B_4_C particles in air atmosphere.

Figure 3 shows the TG and DTG curves of MoSi_2_ particle-modified boron phenolic resin composites with different contents of B_4_C particles in an air atmosphere. The slight weight loss below 200 °C was mainly caused by volatilization of absorbed water while that at 200 °C to 400 °C was mainly due to further dehydration reaction of the residual aldehydes and phenolic monomers in the boron phenolic resin [34]. The strong peaks in the DTG curve at around 750 °C to 900 °C were induced by the violent pyrolysis of resin to produce gases such as CO, CO_2_, C_2_H_6_, H_2_O, etc. [35,36]. The residue yields at 1400 °C were 24.03%, 28.36%, 30.22%, 32.91%, and 30.02%, respectively. It is obvious that the residue yield went high with the increase in contents of B_4_C particles but decreased slightly when the content of B_4_C reached 20 phr. The main reason is that B_4_C react with oxygen at elevated temperatures to produce B_2_O_3_, which is a weight-gaining reaction. However, B_2_O_3_ is easy to volatilize at elevated temperatures, which is a weight-decreasing process. Additionally, the DTG curve and Table 2 show that the greater the content of B_4_C particles, the higher the temperature when the maximum pyrolysis reaction rate is reached. Therefore, 15 phr B_4_C can efficiently increase the thermal stability and residue yield of composites.

### 3.2. Flexural Strength of Composites Treated at Different Temperatures

**Figure 4:**
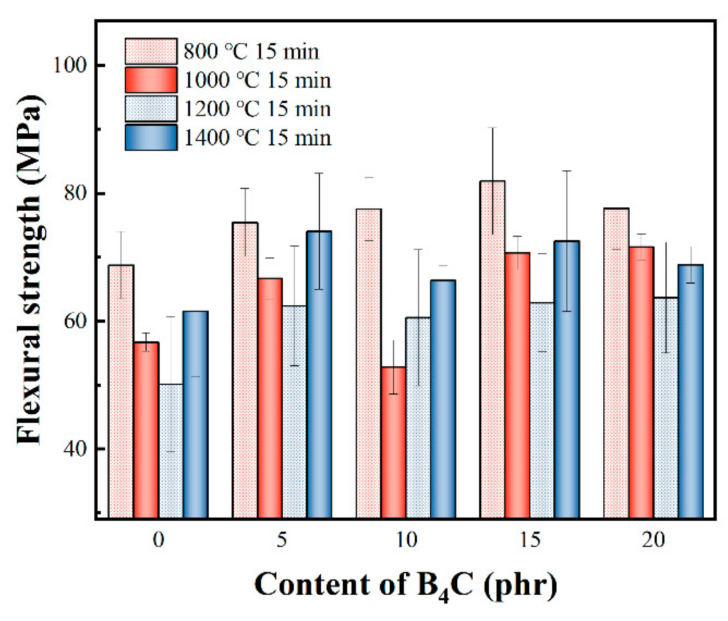
Flexural strength of composites with different contents of B_4_C after treatment at different temperatures.

The influence of B_4_C particulate content on the flexural strength of composites was studied, as shown in Figure 4. After being treated at 800 °C for 15 min, the flexural strength of BP-0 was 68.79 MPa, which increased with increasing B_4_C particles within a certain range. The flexural strength reached its maximum value of 81.91 MPa when 15 phr of B_4_C particles were added; however, it decreased to 77.65 MPa when 20 phr of B_4_C particles were added. The flexural strength dropped with increasing the temperature to 1000 °C and 1200 °C. However, the flexural strength increased abnormally when the temperature was further increased to 1400 °C. To be specific, the flexural strength of BP-0 was 61.58 MPa after being treated at 1400 °C for 15 min, which was much higher than that from being treated at 1000 °C and 1200 °C. Similarly, the same trend is observable for composites with B_4_C particles ranging from 5 phr to 20 phr.

### 3.3. Micromorphology Changes of Composites with Different Contents of B_4_C

**Figure 5:**
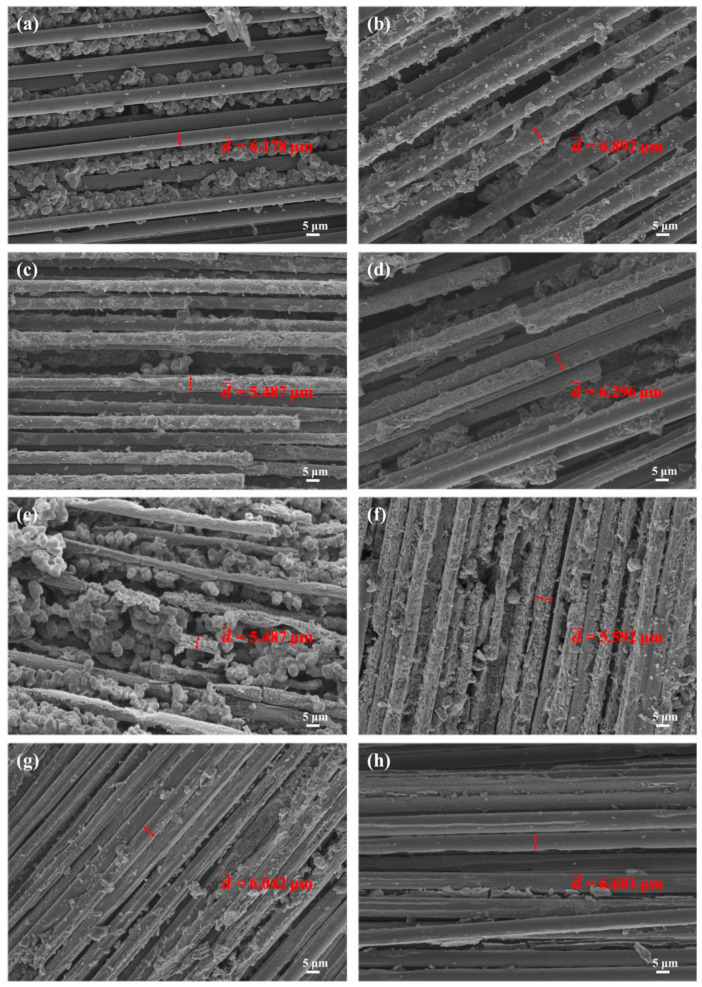
Micromorphology of BP-0 and BP-15 treated at different temperatures for 15 min: BP-0 at (**a**) 800 °C, (**c**) 1000 °C, (**e**) 1200 °C, and (**g**) 1400 °C; BP-15 at (**b**) 800 °C, (**d**) 1000 °C, (**f**) 1200 °C, and (**h**) 1400 °C.

To figure out the reasons for this evolution of flexural strength, SEM analysis was conducted on the surface of the composites after treatment at different temperatures. As shown in Figure 5 and Table 3, the diameter of the carbon fibers exposed for both BP-0 and BP-15 dropped with increasing temperature, ranging from 800 °C to 1200 °C. However, the diameter of carbon fibers in the composites treated at 1400 °C was slightly smaller than that of those treated at 800 °C and much bigger than that of those treated at 1000 °C and 1200 °C. The above findings are consistent with the evolution of flexural strength. That is to say, the carbon fibers in composites treated at 1400 °C were less oxidized than those treated at 1000 °C and 1200 °C, which is incredible.

### 3.4. Phase Evolution of Composites at Elevated Temperatures

**Figure 6:**
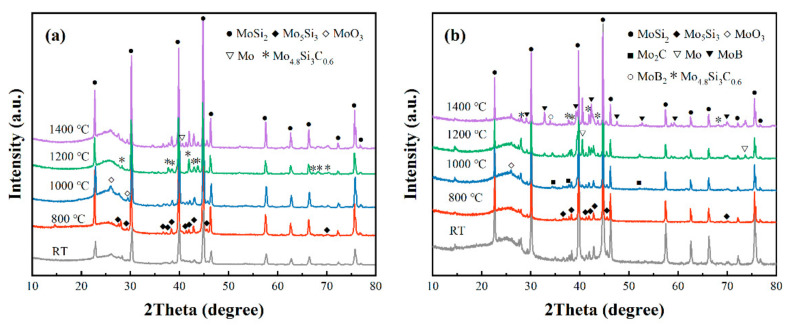
XRD patterns of the residue phases of BP-0 (**a**) and BP-15 (**b**) at different temperatures.

To further find out the reasons for the diameter changes in the composites and explore the phase evolution of composites at elevated temperatures, the composites were subjected to phase analysis after high-temperature treatment. It can be seen from Figure 6 that the main component of both BP-0 and BP-15 at temperatures ranging from room temperature to 1400 °C was MoSi_2_, which indicates that the MoSi_2_ in the composites was relatively stable while the boron phenolic resin itself pyrolyzed intensely at high temperatures. 

When the temperature reached 800 °C, a small amount of MoSi_2_ reacted with oxygen to produce Mo_5_Si_3_ and amorphous SiO_2_ [37,38]. However, due to its amorphous state, no obvious SiO_2_ peak was detected.

5MoSi_2_ + 7O_2_(g) = Mo_5_Si_3_ + 7SiO_2_ (3)

After being treated at 1000 °C, it can be seen in the XRD patterns that the MoO_3_ phase appeared in both BP-0 and BP-15, which was due to the selective oxidation of MoSi_2_. Moreover, MoO_3_ reacted with pyrolytic carbon to form carbides, so the Mo_2_C phase could be found in BP-15.

MoSi_2_ + 3.5O_2_(g) = MoO_3_ + 2SiO_2_ (4)

2MoO_3_ + 7C = Mo_2_C + 6CO(g) (5)

In the XRD pattern of BP-0 at 1200 °C, the Mo_4.8_Si_3_C_0.6_ crystal phase was identified; it is possible that it was generated from the reaction of MoSi_2_ with pyrolytic carbon in an air atmosphere [39,40,41]. At the same temperature, the characteristic peaks of Mo were found in the XRD pattern of BP-15, which may be attributed to the reaction of MoO_3_ with pyrolytic carbon as well.

4.8MoSi_2_ + 0.6C + 6.6O_2_(g) = Mo_4.8_Si_3_C_0.6_ + 6.6SiO_2_ (6)

MoO_3_+ 3C = Mo + 3CO(g) (7)

As the treatment temperature increased to 1400 °C, characteristic peaks of Mo were found in the XRD patterns of BP-0, while Mo_4.8_Si_3_C_0.6_, MoB and MoB_2_ phases were identified in the XRD patterns of BP-15. The occurrence of borides was attributed to the addition of B_4_C. The equations of the chemical reactions involved are as follows [18,42].

MoO_3_ + 0.25B_4_C + 2.75C = MoB + 3CO(g) (8)

MoO_3_ + 0.5B_4_C + 2.5C = MoB_2_ + 3CO(g) (9)

### 3.5. Micromorphology Changes and Phase Evolution of Carbon Fibers Treated at Different Temperatures

To further confirm the positive effect of MoSi_2_ and B_4_C on carbon fiber anti-oxidation, as-received carbon fibers were treated the same as composites and acted as a blank comparison. The treated carbon fibers were characterized using SEM, FT-IR and Raman spectra.

**Figure 7:**
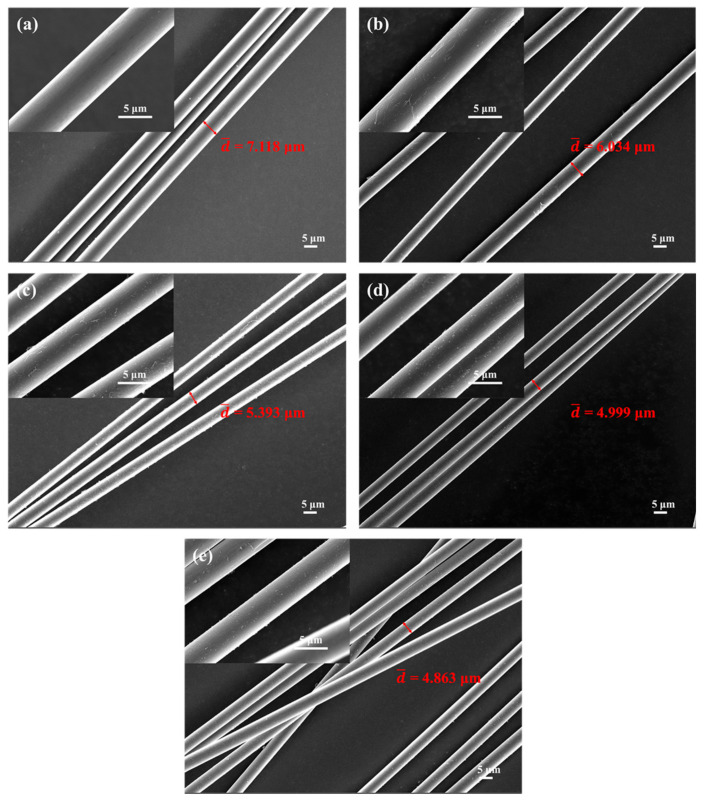
Micromorphology of carbon fibers treated at different temperatures for 15 min: (**a**) room temperature, (**b**) 800 °C, (**c**) 1000 °C, (**d**) 1200 °C, and (**e**) 1400 °C.

Figure 7 shows the changes in the surface morphology of carbon fibers treated at different temperatures. As shown in Figure 7 and Table 3, the diameter of the carbon fibers decreased with increasing treatment temperatures. To be specific, the diameter of carbon fibers at room temperature was about 7.118 μm, decreasing sharply to 4.863 μm after being treated at 1400 °C for 15 min. Obviously, the carbon fibers reacted with oxygen to form CO and/or CO_2_, leading to the decrease in diameter of the carbon fibers.

**Figure 8:**
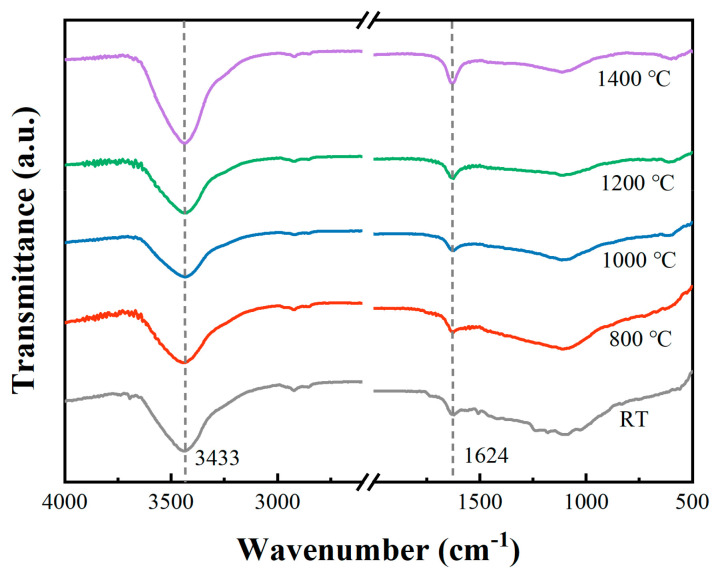
FT-IR spectra of carbon fibers.

FT-IR was used to observe the functional group changes before and after being treated at different temperatures. As shown in Figure 8, the peak attributed to -OH stretching vibration at ~3433 cm^−1^ and the peak associated with C=O stretching vibration at ~1624 cm^−1^ were enhanced with increasing treatment temperatures. The above results indicate that the degree of oxidation of carbon fibers deepened with increasing treatment temperatures, which is consistent with the SEM results.

**Figure 9:**
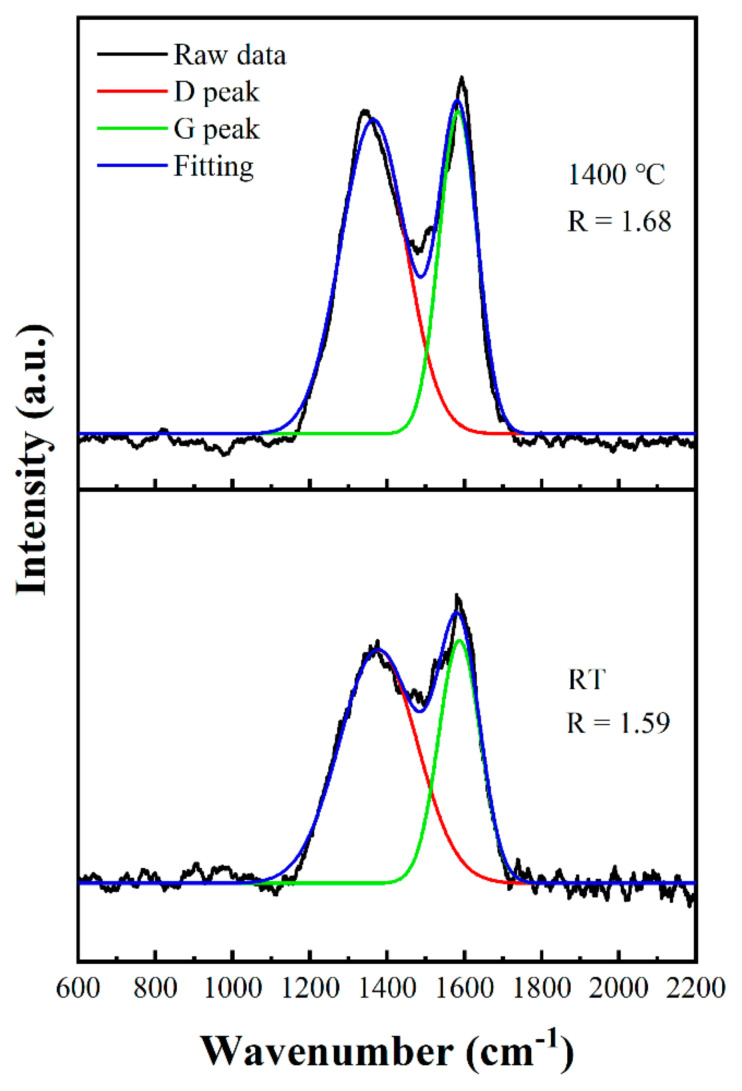
Raman spectra of carbon fibers.

Meanwhile, Raman spectra were used to identify the degree of graphitization of the carbon fibers before and after being treated at 1400 °C (Figure 9). After being oxidized in air at 1400 °C for 15 min, the calculated R value increased from 1.59 to 1.68, indicating that the degree of graphitization decreased after treated at 1400 °C. Raman spectra further confirmed that the degree of oxidation of the carbon fibers deepened as well.

### 3.6. The Ablation Behavior of Composites

#### 3.6.1. The Ablation Properties of Composites under an Oxygen–Acetylene Torch

**Figure 10:**
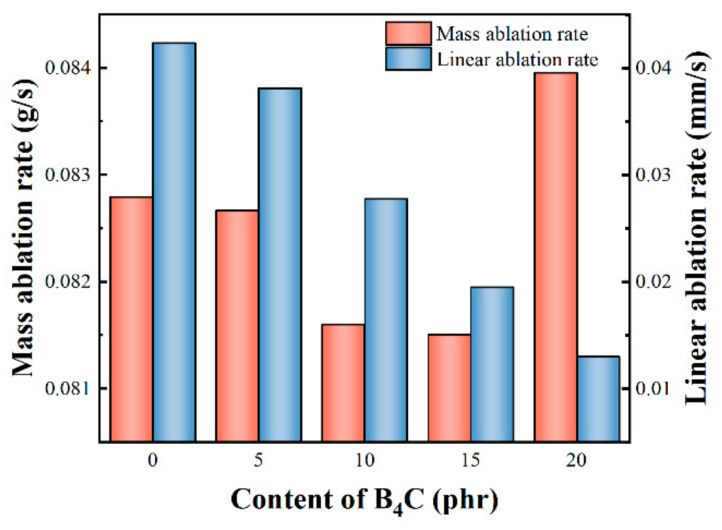
Mass ablation rate and linear ablation rate of composites.

As shown in Figure 10, the linear ablation rate of the composites decreased markedly with increasing B_4_C particles. To be specific, the linear ablation rate was 0.0424 mm/s without B_4_C particles, dropping sharply to 0.013 mm/s with 20 phr B_4_C particles. However, this was not the case for the mass ablation rate. The mass ablation rate was 0.0828 g/s without B_4_C particles, decreasing smoothly with increasing B_4_C particles within a certain range. The mass ablation rate reached its minimum value of 0.0815 g/s with 15 phr B_4_C particles before it went high to 0.0840 g/s with 20 phr B_4_C particles. Two factors may account for this. On the one hand, as described in 3.1, the oxidation reaction of B_4_C is a weight gain reaction while the volatilization of B_2_O_3_ is a weight loss process. On the other hand, the melting temperature of B_2_O_3_ is only about 450 °C. Therefore the molten B_2_O_3_ can easily be washed away from the surface of a composite under an oxygen-acetylene torch with temperatures up to 1900 °C and high velocity airflow, this being a weight loss process as well [43,44,45,46]. Therefore, a compromise between weight-gaining reactions and weight loss processes was reached with 15 phr B_4_C particles, leading to the minimum mass ablation rate. When the B_4_C content increased to 20 phr, more molten B_2_O_3_ was generated on the surface of the composite. However, due to the oxyacetylene torch, a large amount of molten B_2_O_3_ was washed away from the composite surface, far exceeding the amount of oxidation weight gain of B_4_C, resulting in a high mass ablation rate.

#### 3.6.2. Morphology of Ablated Surface

**Figure 11:**
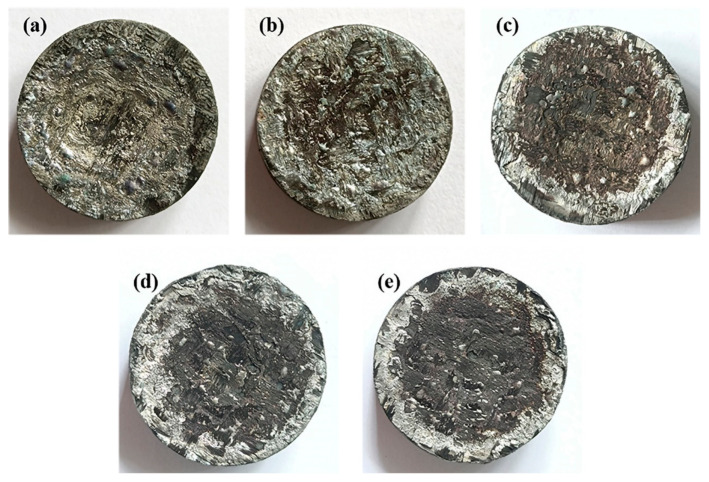
Macroscopic morphology of composites after oxygen–acetylene ablation: (**a**) BP-0, (**b**) BP-5, (**c**) BP-10, (**d**) BP-15, and (**e**) BP-20.

Figure 11 shows the macroscopic morphology of the composites after oxygen–acetylene ablation. It can be seen that there was an obvious pit in the middle of BP-0 after being ablated that shallowed gradually with increasing B_4_C particles, which is consistent with the previous linear ablation rate results. In addition, a white substance occurred and increased on the outer ring of the ablated surface with increasing B_4_C particles.

**Figure 12:**
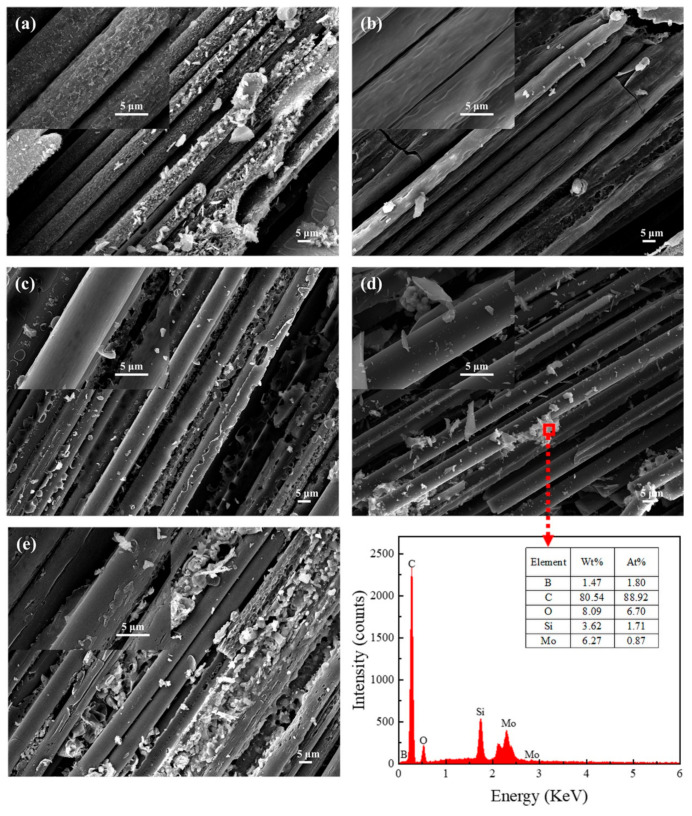
Micromorphology of the ablated surface for composites modified with different B_4_C content: (**a**) BP-0, (**b**) BP-5, (**c**) BP-10, (**d**) BP-15, and (**e**) BP-20. EDS analysis of oxyacetylene ablation sample of BP-15.

Meanwhile, the microstructure of the ablated surface was observed as well (Figure 12). Obviously, there were serious and visible damages, such as holes and grooves, on the surface of the carbon fibers in composites without B_4_C particles. The degree of oxidation of the carbon fibers was greatly relieved with increasing B_4_C particles; that is to say, no obvious holes were observable in BP-10, BP-15 and BP-20.

#### 3.6.3. Phase Composition of Ablated Surface

**Figure 13:**
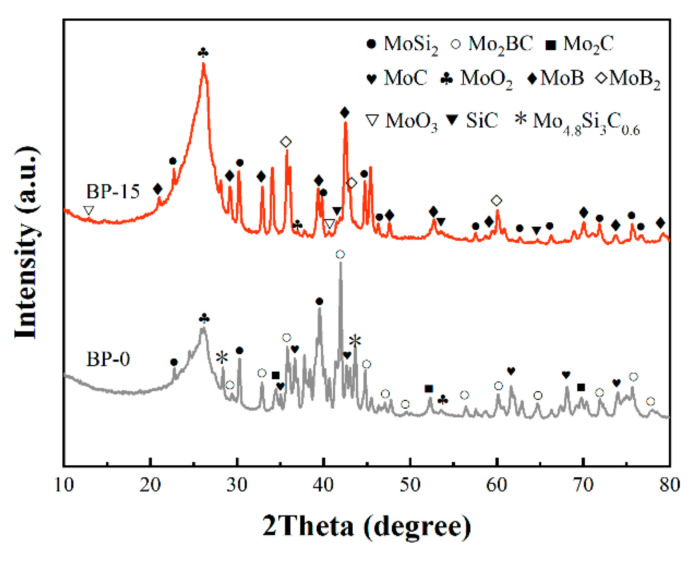
XRD patterns of residues from the ablated surface for BP-0 and BP-15.

To further identify the phase compositions of the ablated surface, the residues scraped off the surface after ablation are subjected to phase analysis by the X-ray diffractometer, as shown in Figure 13. It can be observed that the surface residues of BP-0 were significantly different from those of BP-15. Except for the characteristic peaks of MoSi_2_ that were introduced, the peaks of MoO_2_ were observed in the XRD patterns of BP-0 and BP-15, which are mainly attributed to the oxidation of MoSi_2_ during ablation (reaction (10)). In addition, there were characteristic peaks of MoO_3_ in the XRD pattern of BP-15 (reaction (4)).

MoSi_2_ + 3O_2_(g) = MoO_2_ + 2SiO_2_ (10)

The characteristic peaks of Mo_2_BC, Mo_2_C, MoC and Mo_4.8_Si_3_C_0.6_ can also be seen in the XRD patterns of the BP-0 composites. Among them, Mo_2_C and Mo_4.8_Si_3_C_0.6_ were possibly formed by reaction (5) and reaction (6) respectively [47]. The reactions involved in the generation of Mo_2_BC and MoC are as follows [42].

2MoSi_2_ + CO(g) + 2.75O_2_(g) + 0.5B_2_O_3_ = Mo_2_BC + 4SiO_2_ (11)

2MoSi_2_ + C + 3.25O_2_(g) + 0.5B_2_O_3_ = Mo_2_BC + 4SiO_2_ (12)

MoSi_2_ + CO(g) + 1.5O_2_(g) = MoC + 2SiO_2_ (13)

MoSi_2_ + C + 2O_2_(g) = MoC + 2SiO_2_ (14)

Besides the characteristic peaks of MoO_2_ and MoO_3_, the characteristic peaks of MoB, MoB_2_ and SiC can also be seen in the XRD pattern of BP-15. A possible reason for this is that MoSi_2_ undergoes oxidation reactions (4), (10) at a higher temperature, after which reactions (8), (9), (15), and (16) occur [48].

MoO_2_ + 0.25B_4_C + 1.75C = MoB + 2CO(g) (15)

MoO_2_ + 0.5B_4_C + 1.5C = MoB_2_ + 2CO(g) (16)

As for SiC, it was possibly derived from the reaction of SiO_2_ and PyC [12,47].

SiO_2_ +3C = SiC + 2CO(g) (17)

The thermodynamic parameters of the reactions involved in this paper are shown in Table A1.

### 3.7. Effect of MoSi_2_ and B_4_C on Carbon Fiber Oxidation in Composites

Combined with the above-mentioned results and discussions, MoSi_2_ and B_4_C particles possess a positive effect on carbon fiber an-oxidation in composites. On the one hand, MoSi_2_ and B_4_C particles can react with oxygen and other oxygen-containing gases, such as CO. In other words, MoSi_2_ and B_4_C particles consume a lot of oxygen around the composites, thus the oxidation reaction of carbon fibers in composites is mitigated. On the other hand, molten B_2_O_3_ derived from B_4_C acts as an oxygen barrier so that an amount of oxygen can be blocked outside the composites, which mitigates the oxidation degree of carbon fibers in the composites as well. In addition, multiphase ceramics derived from MoSi_2_, such as MoB, MoB_2_, Mo_2_C and Mo_4.8_Si_3_C_0.6_, play the roles of oxygen barriers as well. Therefore, BP-15, which is composed of carbon fibers, boron phenolic resin and MoSi_2_ and B_4_C particles, exhibits excellent high temperature behavior.

## 4. Conclusions

In this work, the thermal stability, flexural strength, phase composition, microstructure, and ablation resistance of ceramizable composites were studied, and the oxidation behavior of carbon fibers in composite materials was demonstrated. 

The results indicate that the incorporation of B_4_C improves the performance of composites at high temperatures. TG-DTG shows that the residual weight rate of composites with 15 phr B_4_C(BP-15) sufficiently increased from 23.03% to 32.91% compared with the composites without B_4_C(BP-0). After being treated at 1400 °C for 15 min, the flexural strength of BP-15 increased by 17.79% compared with BP-0. The incorporation of B_4_C particles effectively improved the ablation performance of the composites. Compared with BP-0, the line ablation rate and mass ablation rate of BP-15 were reduced by 53.96% and 1.56%, respectively. Compared with BP-0, the line ablation rate and mass ablation rate of BP-15 were reduced by 53.96% and 1.56%, respectively. In addition, MoSi_2_ and B_4_C particles have a positive effect on the oxidation of carbon fibers in composite materials. The oxidation degree of carbon fibers was greatly relieved with increasing B_4_C particles. After treatment at 1400 °C, the diameter of the as-received carbon fiber was reduced by 31.68%, while the diameter of carbon fiber in BP-0 and BP-15 decreased by 15.12% and 6.14%, respectively.

According to the morphology and XRD analysis of the composites, we found that MoSi_2_-derived complex-phase ceramics (MoB, MoB_2_, Mo_2_C, Mo_4.8_Si_3_C_0.6_, etc.) with high melting points were formed at high temperatures. Liquid B_2_O_3_ and MoSi_2_-derived complex-phase ceramics played a role of an oxygen barrier, effectively mitigating the oxidation degree of the carbon fibers and enhancing the performance of composites at high temperatures.

## Figures and Tables

**Table 1:** Formula of ceramizable boron phenolic resin solution.

Samples	Boron Phenolic Resin Solution (phr)	MoSi_2_ (phr)	B_4_C (phr)
BP-0	100	65	0
BP-5	100	65	5
BP-10	100	65	10
BP-15	100	65	15
BP-20	100	65	20

**Table 2:** Thermal decomposition characteristics of composites.

Samples	^1^ Tmax/°C	Residue Yield/%		
		500 °C	1000 °C	1400 °C
BP-0	755.2	93.20	50.78	24.03
BP-5	813.4	92.11	54.02	28.36
BP-10	885.0	93.44	66.31	30.22
BP-15	886.0	93.93	69.92	32.91
BP-20	892.4	93.79	66.65	30.02

^1^: Tmax is the temperature at which the rate of thermal weight loss is maximum.

**Table 3:** Diameter changes of carbon fibers treated at different temperatures.

Treatment Temperature	$\bar{d}$/μm		
	Carbon Fibers	BP-0	BP-15
RT	7.118	7.118	7.118
800 °C	6.034	6.178	6.892
1000 °C	5.393	5.487	6.296
1200 °C	4.999	5.097	5.592
1400 °C	4.863	6.042	6.681

## Data Availability

The data presented in this study are available on request from the corresponding author.

## Appendix A

**Table A1:** Thermodynamic parameters ^2^ of reactions.

**Reaction**	**∆G (kJ/mol)**							
	**800 °C**	**1000 °C**	**1200 °C**	**1400 °C**	**1600 °C**	**1800 °C**	**2000 °C**	**2200 °C**
(3)	−4708.499	−4478.956	−4252.360	−4028.161	−3805.981	−3588.251	−3377.260	−3168.831
(4)	−1781.391	−1675.767	−1572.219	−1470.295	−1369.734	−1271.108	−1174.976	−1080.064
(5)	−344.272	−525.977	−703.651	−878.027	−1049.531	−1218.490	−1385.158	−1549.733
(7)	−144.394	−234.337	−322.212	−408.403	−493.150	−576.646	−659.053	−740.511
(8)	−242.457	−330.223	−415.840	−499.675	−581.943	−662.808	−742.401	−820.826
(9)	−206.397	−296.627	−385.521	−473.544	−560.980	−648.041	−734.887	−821.643
(10)	−1699.199	−1597.309	−1496.734	−1397.354	−1299.107	−1202.741	−1108.966	−1016.649
(13)	−1128.581	−1042.883	−957.937	−873.603	−789.778	−707.155	−626.376	−546.231
(14)	−1335.313	−1267.079	−1199.388	−1132.122	−1065.191	−999.301	−935.104	−871.398
(15)	−117.916	−184.486	−249.874	−314.098	−377.157	−439.029	−499.683	−559.075
(16)	−81.856	−150.890	−219.554	−287.967	−356.194	−424.262	−492.169	−559.892
(17)	240.864	173.222	106.285	39.969	−25.786	−90.633	−154.249	−217.232
^2^: Thermodynamic parameters come from the HSC Chemistry software.								
